# Phenotypes and outcomes in middle-aged patients with diabetic foot ulcers: a retrospective cohort study

**DOI:** 10.1186/s13047-020-00386-z

**Published:** 2020-05-15

**Authors:** Tao Tong, Cailian Yang, Wenqing Tian, Zhiping Liu, Bo Liu, Jun Cheng, Qingfeng Cheng, Bo Zhou

**Affiliations:** 1 Department of Endocrinology, Xiangyang No.1 People’s Hospital, Affiliated to Hubei University of Medicine, Xiangyang, 441000 China; 2 Department of Endocrinology, The First Affiliated Hospital of Chongqing Medical University, Chongqing, China; 3 Department of Burns & Plastic Surgery, The First Affiliated Hospital of Chongqing Medical University, Chongqing, China; 4 Department of Vascular Surgery, The First Affiliated Hospital of Chongqing Medical University, Chongqing, China

**Keywords:** Diabetic foot ulcer;phenotype, Outcome, Middle-aged

## Abstract

**Background:**

Although ageing could increase the risk of delayed healing in diabetic foot ulcers (DFUs) patients, data from middle-aged patients remains greatly limited. The purpose of this study was to explore the clinical phenotypes, outcomes and predictive factors of DFU in middle-aged patients.

**Methods:**

A retrospective cohort study conducted with 422 consecutive inpatients with DFUs who visited our hospital between May 2010 and September 2017; participants were recruited and assigned according to age to either the middle-aged group or the elderly group. The Demographics, ulcer characteristics, comorbidities and diabetes complications, laboratory tests, socioeconomic data and final outcomes were collected. Moreover, predictive factors of adverse outcomes in middle-aged DFUs patients were assessed.

**Results:**

Middle-aged patients were more likely to have worse lifestyle and glucose control, were more likely to have microangiopathy as a complication, and tended to have larger and deeper ulcers; however, these patients also had higher rates of healing and lower rates of mortality and major amputaion than elderly patients. Severe infection,living alone,current smoking cigarettes, and having a high white blood cell count were independent risk factors for adverse outcomes in middle-aged patients.

**Conclusions:**

DFUs are relatively common in middle-aged patients with diabetes, and these patients have unique clinical phenotypes and risk profiles. Nonetheless, further investigation is needed to clarify whether intervention targeting these easily recognizable risk factors can improve healing and survival rates in middle-aged DFU patients.

## Background

Diabetic foot ulcer (DFU) is the most frequent cause of hospitalization among diabetic patients, and lower extremity amputation is the most feared consequence of this disorder, with disastrous effects on patient health and quality of life [1]. Previous studies identified that the healing of foot ulcers is a complex,dynamic and multifactorial process that involves the interaction of diabetes complications, ulcer characteristics, and malnutrition, and the complexities of the healing process can be compounded by the patient’s social-economic status, level of self-care and age [2–8]. Although preliminary studies had suggested that ageing could increase the risk of delayed healing in DFU patients [4, 6, 7], data from middle-aged patients remains greatly limited. Moreover, the overall prevalence of diabetes and early-onset diabetes has sharply increased in recent decades in both China and other developing countries [9, 10], and it is likely that the number of DFUs among middle-aged patients aged 45–64 years will similarly increase. More importantly, DFUs in middle-aged working adults can cause unemployment, disability, and even death in the prime of life, contributing to increased family, social,and health care burdens [11, 12]. Therefore, it is very important to understand the clinical phenotypes of DFUs in middle-aged patients to inform the design of a new approach to diminishing the adverse outcomes of DFU in middle-aged patients.

To our knowledge, there has been no systematic and comprehensive study conducted on the clinical features of and predictors of outcomes in middle-aged DFU patients. Thus, the aims of the present study were as follows: 1) to explore the phenotypes and outcomes of DFUs in middle-aged patients, comparing those phenotypes and outcomes with those in the elderly and 2) to assess the variables that best predict poor outcomes in middle-aged patients.

## Methods

### Study design and participants

This single-centre retrospective cohort study was conducted in the First Teaching Hospital of Chongqing Medical University, a tertiary care setting with a multidisciplinary foot care team. A total of 422 consecutive inpatients with DFU who visited our hospital between May 2010 and September 2017 were recruited. DFU was defined as a full-thickness wound, skin necrosis or gangrene below the ankle including peripheral neuropathy or peripheral arterial disease in patients with diabetes [13]. Excluded patients were those who had malignancies, autoimmune disease, severe liver disease, venous ulcers, or dementia as well as those younger than 18 years old.

### Age

The chronological age greater than or equal to 65 years has been accepted as the definition of “elderly” or “older person” in most developed countries. In addition, a large number of the studies that have reported on the clinical characteristics and outcomes of patients with diabetes or diabetic foot used a cut-off of 65 years to distinguish between elderly and non-elderly patients [14–16]. Thus, the patients in our study were divided into two groups based on age, and 65 years was chosen as the age used to distinguish between middle-aged patients (aged 45–64 years) and elderly patients (age ≥ 65 years).

### Datacollection and definitions

We collected basic data for all patients by reviewing their electronic medical records and conducting structured interviews. We obtained the following data from admission records: (1) demographics, such as age, gender, body mass index (BMI), history of current smoking and alcohol consumption, type of diabetes,treatment regimen and duration of diabetes; (2) laboratory test results, including haemoglobin (Hb), serum albumin (ALB), fasting plasma glucose (FPG), glycated haemoglobin (HbA1c), white blood cell count (WBC), estimated glomerular filtration rate (eGFR) [17], total cholesterol (TC), triacylglycerol (TG), low-density lipoprotein cholesterol (LDL-C), and high-density lipoprotein cholesterol (HDL-C); (3) complications associated with diabetes such as diabetic retinopathy (DR), nephropathy (DN), and peripheral neuropathy (DPN); (4) other comorbidities such as hypertension,coronary heart disease (CHD), lower extremity peripheral arterial disease (PAD), and stroke; (5) ulcer characteristics, such as ulcer duration (defined as the time elapsed between the onset of symptoms and hospital admission) and the size, depth and infection status of the ulcer; and (6) treatment of the foot ulcer, including debridement, antibiotic choices and revascularization,collected through the treatment episode.

In this study, PAD was classified into the following three grades based on the perfusion of the limbs: grade 1 involved no PAD, grade 2 displayed symptoms or signs of PAD but not of critical limb ischaemia (CLI), and grade 3 involved CLI [18, 19]. The area of the ulcer was estimated by multiplying the largest diameter by the second largest diameter that was measured perpendicular to the first diameter, and the area was expressed in cm^2^. The depth of the ulcer was categorized as follows:grade 1(ulceration extending to subcutaneous tissue),grade 2(ulceration involving the joint capsule or tendon), and grade 3 (ulceration extending into the bone or within a joint) [18]. The diagnosis of infection was based principally on the presence or absence of signs and symptoms of inflammation,the presence of secretions, and the results of laboratory and imaging tests when admitted. Infections were classified into 4 grades according toperfusion-extent-depth-infection-sensation (PEDIS) system [18, 20].

The data on socioeconomics, foot care and behavioural characteristics were collected as in a previous study [21],these were as follows: (1) socioeconomic status, as represented by medical insurance status, income level, cohabitation status, housing conditions, and education level and (2) foot care and behavioural characteristics,including walking barefoot, inspecting the foot routinely, knowing the danger signs of foot lesions and the relevant diabetic foot care, and visiting the diabetic clinic.

### Study main outcome

In the present study, all DFU patients were followed for 6 months or until death. Generally, the endpoints of the DFU were healed (defined as a continuous viable epithelial covering over the entire previously open wound), unhealed (defined as incomplete re-epithelialization of the wound), amputation (including major and minor amputations) or death (all-cause mortality) [7, 22]. The primary composite endpoint of our study was a combined desired outcome (healed) and adverse outcome (unhealed, amputation, and death). The secondary endpoint was amputation or death, respectively.

### Statistical analysis

Categorical data are expressed as numbers, and the χ^2^ test or Fisher’s exact test was used to evaluate the differences in distribution. The normally distributed continuous variables are expressed as the means ± SD, and Student’s t test was used to assess the differences. Non-normally distributed variables are expressed as the medians with interquartileranges, and the nonparametric Mann–Whitney U test was used to assess the differences. Furthermore,a multivariable logistic regression analysis was performed to assess which variables were independently associated with adverse outcomes in the middle-aged patients. The associations are presented as odds ratios (ORs) with their corresponding 95% confidenceintervals (95% CIs). We conducted all analyses using SPSS version 19.0 statistical software (SPSSInc.,Chicago, IL, USA); *P* < 0.05 was considered statistically significant.

## Results

### Characteristics of the study population

During the study period,a total of 445 patients were recruited. However, 23 patients had failed to including in our study according to exclusion criteria:2 patients with malignancies, 2 patients with autoimmune disease, 1 patient with severe liver disease, 15 patients with venous ulcers,and 3 patients with dementia. So,422 patients (mean age 66.2 years), including 175(41.4%) middle-aged patients and 247(58.6%) elderly patients, were eligible for analysis. Of these patients, 265 (62.8%)were men, 128(30.3%) current consumed alcohol, 149(35.3%) had a history of current smoking, 414 (98.4%) had type 2 diabetes, and 230 (54.5%)used insulin; the mean duration of diabetes was 9.7 ± 7.7 years, and the mean duration of DFU was 57.6 ± 78.6 days (Table 1).

**Table 1:** Baseline characteristics of diabetic foot ulcer patients in the two age groups

	All (***N*** = 422)	Middle-aged (***N*** = 175)	Elderly (***N*** = 247)	P
**Demographics**				
Age (years)	66.2 ± 12.0	54.5 ± 8.0	74.4 ± 6.1	**<0.001**
Gender (male/female)	265/157	116/59	149/98	0.212
BMI (kg/m^2^)	22.6 ± 2.9	22.4 ± 3.1	22.8 ± 2.8	0.190
Alcohol consumption (yes/no)	128/294	67/108	61/186	**0.004**
Smoking habit (yes/no)	149/273	76/99	73/174	**0.004**
**Diabetic history**				
Diabetes type (type1/type 2)	8/414	8/167	0/247	**0.001**
Diabetic duration (years)	9.7 ± 7.7	7.9 ± 6.2	11.0 ± 8.4	**<0.001**
Treatment: no treatment/oral drugs/insulin	32/160/230	22/59/96	12/101/134	**0.027**
**Laboratory test**				
Fastingplasma glucose (mmol/L)	11.4 ± 5.4	12.2 ± 6.0	10.9 ± 4.9	**0.017**
HbA1c (%)	9.2 ± 2.4	9.6 ± 2.8	8.2 ± 2.1	**0.003**
Haemoglobin (g/l)	115 (102–129)	118 (100–129)	114 (103–129)	0.894
Serum albumin (g/l)	34.3 ± 6.2	34.1 ± 6.7	34.5 ± 5.8	0.512
White blood cells (10^3^/l)	8.7 ± 4.4	8.9 ± 5.1	8.57 ± 3.8	0.482
eGFR (mL/min/1.73 m^2^)	74.9 ± 28.8	86.7 ± 30.2	66.5 ± 24.5	**<0.001**
Total cholesterol (mmol/L)	4.0 ± 1.1	4.0 ± 1.0	4.1 ± 1.0	0.325
Triglycerides (mmol/L)	1.4 ± 0.7	1.3 ± 0.7	1.4 ± 0.7	0.194
LDL cholesterol (mmol/L)	2.3 ± 0 .8	2.3 ± 0 .9	2.3 ± 0.8	0.377
HDL cholesterol (mmol/L)	1.1 ± 0 .3	1.1 ± 0 .4	1.1 ± 0 .3	0.686
**Comorbidities**				
Hypertension (yes/no)	273/149	89/86	184/63	**<0.001**
Coronary heart disease (yes/no)	69/353	16/159	53/194	**0.001**
History of stroke (yes/no)	44/378	6/169	38/209	**<0.001**
**Diabetic complication**				
Nephropathy (yes/no)	176/246	68/107	108/139	0.318
Peripheral artery disease (1/2/3)	211/127/84	120/36/19	91/91/65	**<0.001**
Retinopathy (yes/no)	125/297	64/111	61/186	**0.008**
Peripheral neuropathy (yes/no)	313/109	139/36	174/73	**0.042**
**Ulcer characteristics**				
Duration of ulcer (days)	57.6 ± 78.6	54.5 ± 77.2	56.3 ± 79.7	0.815
Extent (cm^2^)	8.1 ± 16.0	10.5 ± 21.5	6.4 ± 10.30	**0.019**
Depth (1/2/3)	97/249/76	43/91/41	54/158/35	**0.021**
Infection (1/2/3/4)	18/245/130/29	6/108/49/12	12/137/81/17	0.584
Debridement (yes/no)	155/267	67/108	88/159	0.609
Antibiotics (≤ 1/≥ 2)	247/175	92/83	155/92	**0.045**
Revascularization (yes/no)	47/357	10/165	37/210	**0.003**

### Baseline characteristics of the two groups

The baseline characteristics of DFU in different age groups are shown in Table 1. Compared with the elderly patients, the middle-aged patients were more likely to have a history of smoking and alcohol consumption, a shorter duration of diabetes, and higher levels of FPG and HbAlc; they were also more likely to have DR and DPN as complications. In contrast, middle-aged patients were less likely to have hypertension or a history of CHD and stroke, and their levels of kidney function impairment and PAD were significantly lower than those in the elderly group. However, gender, BMI, laboratory test results (levels of Hb and ALB, WBC, and lipid profile) and the incidence of diabetic nephropathy were not significantly different between the two groups. Additionally, the middle-aged patients tended to have larger and deeper ulcers,were more likely to be using at least 2 types of antibiotics, and were less likely to be eligible for revascularization of the lower limb than the elderly patients. There was no significant difference in debridement, ulcer duration or the severity of infection between the two groups (all *p* value > 0.05).

The data on the socioeconomic status, foot care and behavioural characteristics of two groups are shown in Table 2. Most patients, regardless of age, had medical insurance and a high to average income but had inadequate knowledge of the signs indicating dangerous foot lesions and poor performance of foot inspection; most variables were not significantly different between the two groups, except education level (all *p* value > 0.05).

**Table 2:** Comparison of socioeconomic status, foot care and behavioural characteristics of the two groups

	All (***N*** = 422)	Middle-aged (***N***=175)	Elderly (***N*** = 247)	p
**Social-economic status**				
Medical insurance (yes/ no)	310/112	128/47	182/65	0.901
Income level (high/moderate/low)	178/176/68	69/78/28	109/98/40	0.569
Live alone (yes/no)	32/390	14/161	18/229	0.901
Housing conditions (good/moderate/bad)	148/255/19	61/104/10	87/151/9	0.598
Educationallevel (primary school /secondaryschool/university)	198/168/56	51/85/39	147/83/17	**<0.001**
**Foot care and behavioural characteristics**				
Walking barefoot (yes/no)	51/371	26/149	25/222	0.139
Performanceoffoot inspection (usually/sometimes/seldom)	121/247/54	55/92/28	66/155/26	0.082
Knowledge of foot lesion danger signals (yes/ no)	168/254	77/98	91/156	0.331
Foot education received (yes/ no)	375/47	152/23	223/24	0.141
Diabetic clinic visits(> 2/1–2/< 1 per year)	113/267/42	42/114/19	71/153/23	0.592

### Outcomes and the predictive factors of DFU in middle-aged patients

The outcomes in our study are shown in Table 3; 65.9% of all DFU patients had a favourable outcome, and the rates of amputation and death during the follow-up period were 16.8 and 15.9%, respectively. The middle-aged patients had better healing rates than those of the elderly patients (74.3% vs. 59.9%, *p* = 0.002). Similar results were also observed for mortality and major amputation; middle-aged DFU patients had a lower rate of all-cause mortality and major amputation than that of the elderly patients (10.9% vs. 19.4%, *p* = 0.018, 0.6% vs. 5.7%, *p* = 0.005). However,the rate of all-amputation and minor amputation between these two groups was not significantly different(all *p* value > 0.05).

**Table 3:** The outcomes the of diabetic foot ulcers in the two groups

	All (***N*** = 422)	Middle-aged (***N*** = 175)	Elderly (***N*** = 247)	P
**Primary outcome, n (%)**				
Desired outcome	278 (65.9%)	130 (74.3%)	148 (59.9%)	**0.002**
**Secondary outcome, n (%)**				
All-Amputation	71 (16.8%)	26 (14.9%)	45 (18.2%)	0.363
Major-amputation	15 (3.5)	1 (0.6)	14 (5.7)	**0.005**
Minor-amputation	56 (13.3)	25 (14.3)	41 (12.6)	0.353
All-cause mortality	67 (15.9%)	19 (10.9%)	48 (19.4%)	**0.018**

To reduce unnecessary disability and premature death, we further explored the factors that were predictive of adverse outcomes in middle-aged DFU patients. Logistic regression analysis was performed with adverse outcome as the dependent variableand baseline categories that were significant in the univariate analysis (*p* < 0.1) as independent variables. The final results showed that severe infection (OR 6.52, 95% CI 3.14–13.55; *P* < 0.001), living alone (OR 5.94, 95% CI 1.55–22.74; *P* = 0.014), smoking (OR 2.64, 95% CI1.11–6.28; *P* = 0.029) and increased WBC counts (OR 1.14, 95% CI 1.04–1.25; *P* = 0.005) were independent risk factors of adverse outcomes in middle-aged patients (Table 4).

**Table 4:** Regression analysis assessing the associations between the risk factors and adverse outcomes in middle-aged groups

	Odds Ratio	95% CL of Odds Ratio	P
Infection (1/2/3/4)	6.52	3.14–13.55	**<0.001**
Live alone (yes/no)	5.94	1.55–22.74	**0.014**
Smoking (yes/no)	2.64	1.11–6.28	**0.029**
White blood cells (10^3^/l)	1.14	1.04–1.25	**0.005**

## Discussion

DFUs, a severe complication of diabetes, tend to heal poorly and require long and intensive treatment, and they eventually lead to a high risk of amputation and even death. Abundant evidence has demonstrated that early recognition of diabetic foot problems and a coordinated intervention with a multidisciplinary foot care team may significantly improve patient outcomes [23, 24]. Although previous studies revealed that age, an easily measured risk factor, was strongly associated with the risk of amputation and death in patients with DFU [6, 7, 25], the data on the phenotypes and outcomes in middle-aged patients were limited. The present study, to the best of our knowledge, was the first to show that middle-aged patients with DFUs made worse lifestyle choices, such as smoking and consuming alcohol, and had worse glucose control; they also had more severe ulcers and were more likely to have the complications of microangiopathy than elderly patients. However, these patients eventually had better healing rates and a lower risk of major amputation and mortality.

Many studies have noted significant discrepancies in clinical characteristics and coronary angiography results between middle-aged patients with premature myocardial infarction and elderly patients [26–28], but little evidence has emerged regarding the clinical phenotypes of DFUs in non-elderly patients. This study showed that the DFUs of middle-aged patients were larger and deeper than those of elderly patients. The mechanism causing more severe ulcers in these patients remains unknown, which might be partly explained by following two aspects. Previous studies had suggested that those patients with DPN and DR may experience a delay in detecting foot problems and exhibit poor self-management of the wound because of their loss of protective sensation and their poor vision, resulting in a greater severity of the ulcer by the time they visit a doctor [20, 21, 29]. Moreover, long-term hyperglycaemia and smoking may weaken immunity and impair the functioning of inflammatory cells that are important to bactericidal activity [1, 30, 31], thus further increasing the ulcer size and depth. The middle-aged patients with DFU, despite experiencing severe DFUs, had higher rates of healing and lower rates of mortality and major amputation. The reason for these better outcomes in middle-aged patients is not yet clear but might be partly explained by the lower incidence of PAD and the higher eGFR values among these patients. The results of this and other studies have shown that younger subjects have more adequate blood supply to their lower limbs than older subjects, and that greater blood supply is vital for tissue repair and regeneration and combating ulcer infections [32]. On the other hand, Zubair et al. [33] found that DFU healing was worse in patients with impaired renal function than in those who had normal renal function. In addition to other biological factors, ageing itself is characterized by the degeneration of organ function, impaired immunity, and a decreased ability to cope with external stress and to regenerate granulation tissue [34].

The proportion of middle-aged patients with DFUs far exceeded the amount expected based on their relatively young age and short duration of diabetes. Furthermore, DFUs in these patients might lead to decreased social activities, anxiety and depression, and even suicide. Therefore, it was crucial to clarify the risk factors associated with adverse outcomes in these patients. Our findings have suggested four easily recognizable and modifiable risk factors that contribute to poor outcomes in these patients, namely, severe infections, solitary living conditions, cigarettes and increased WBC counts. It is nearly universally agreed among researchers that more severe infections are correlated with poorer outcomes in DFU patients [35, 36]. In addition, amputation and mortality in DFU patients were reduced by early identification of infection and application of antimicrobial therapy. Furthermore, this study also revealed that 43.4% of middle-aged patients had a history of smoking, and the risk of adverse outcomes for patients who smoked was 2.6 times higher than that of those who had never smoked. Similarly, a prospective cohort study with Canadian patients with type 2 diabetes demonstrated that patients who smoked had a risk of developing foot gangrene or requiring amputation that was 4.2 times higher than that of those who did not smoke [15], implying that smoking cessation may be critical for the improvement of the prognosis of DFUs. Although numerous clinical studies found an independent relationship between living alone and patient outcomes following myocardial infarction [37–39], the relation between living alone and DFU prognosis remains to be clarified. Yu et al. [36] failed to find any significant relationship between living alone and DFU outcomes in a larger cohort study of 669 individuals with an average age of 64 years. However, our results showed an independent positive association between living alone and DFU outcomes in patients with an average age of 54.58 years. This discrepancy is likely due to differences in phenotypes at different ages. Thus, more studies are needed in the future to clarify the relationship between living alone and DFU outcomes.

There are some limitations of the current study. First, the study was based in a single centre, limiting its generalizability; therefore, additional large-scale research is needed. In addition, retrospective surveys have inherent deficiencies. A prospective intervention study is needed to establish the direction of causality. Finally, standardized diabetic foot self-care is involved in multiple aspects of DFU outcomes, but the majority of variables in this study were based on the prevention of high-risk foot ulcers and not ulcer care; the relationship between foot self-care and DFU prognosis needs to be clarified by investigating other variables.

## Conclusions

In conclusion, DFU is relativly common in middle-aged patients with diabetes,and these patients have unique clinical characteristics, such as deeper and larger ulcers, worse glucose control, more smoking, more alcohol consumption, and more microangiopathy involvement, but ultimately have better healing rates and alower risk of major amputation and mortality. Although severe infections,solitary living conditions,cigarettes, and increased WBC counts,were independent predictors of adverse outcome in middle-aged patients, further investigation is needed to clarify whether intervention regarding these modifiable risk factors could improve healing and survival rates in these DFU patients.

## Data Availability

Please contact author for data requests.

## Abbreviations

DFU: Diabetic foot ulcer
BMI: Body mass index
HbA1c: Glycated haemoglobin
eGFR: Estimated glomerular filtration rate
Hb: Haemoglobin
ALB: Albumin
FPG: Fasting plasma glucose
WBC: White blood cell
TC: Total cholesterol
TG: Triacylglycerol
LDL-C: Low-density lipoprotein cholesterol
HDL-C: High-density lipoprotein cholesterol
CLI: Critical limb ischaemia
DR: Diabetic retinopathy
DN: Diabetic nephropathy
DPN: Diabetic peripheral neuropathy
CHD: Coronary heart disease
PAD: Peripheral arterial disease
OR: Odds ratio
SPSS: Statistical product and service solutions
PEDIS: Erfusion extent depth infection sensation

## References

[1] Thewjitcharoen Y, Krittiyawong S, Porramatikul S, Parksook W, Chatapat L, Watchareejirachot O (2014). Outcomes of hospitalized diabetic foot patients in a multi-disciplinary team setting: Thailand's experience. J Clin Transl Endocrinol.

[2] Miller W, Berg C, Wilson ML, Heard S, Knepper B, Young H (2019). Risk Factors for Below-the-Knee Amputation in Diabetic Foot Osteomyelitis After Minor Amputation. J Am Podiatr Med Assoc.

[3] Weng C, Coppini DV, Sonksen PH (2000). Geographic and social factors are related to increased morbidity and mortality rates in diabetic patients. Diabet Med.

[4] Costa RHR, Cardoso NA, Procopio RJ, Navarro TP, Dardik A, de Loiola Cisneros L (2017). Diabetic foot ulcer carries high amputation and mortality rates, particularly in the presence of advanced age, peripheral artery disease and anemia. Diabetes Metab Syndr.

[5] Jones PJ, Bibb RJ, Davies MJ, Khunti K, McCarthy M, Fong DTP, et al. A Fitting Problem: Standardising Shoe Fit Standards to Reduce Related Diabetic Foot Ulcers. Diabetes Res Clin Pract. 2019. 10.1016/j.diabres.2019 05.017 PMID:31128134. 10.1016/j.diabres.2019.05.017 31128134

[6] Nather A, Bee CS, Huak CY, Chew JLL, Lin CB, Neo S (2008). Epidemiology of diabetic foot problems and predictive factors for limb loss. J Diabetes Complications.

[7] Martins-Mendes D, Monteiro-Soares M, Boyko EJ, Ribeiro M, Barata P, Lima J (2014). The independent contribution of diabetic foot ulcer on lower extremity amputation and mortality risk. J Diabetes Complications.

[8] Gau B-R, Chen H-Y, Hung S-Y, Yang H-M, Yeh J-T, Huang C-H (2016). The impact of nutritional status on treatment outcomes of patients with limb-threatening diabetic foot ulcers (vol 30, pg 138, 2016). J Diabetes Complications.

[9] Whiting DR, Guariguata L, Weil C, Shaw J (2011). IDF Diabetes Atlas: Global estimates of the prevalence of diabetes for 2011 and 2030. Diabetes Res Clin Pract.

[10] Hu C, Jia W (2018). Diabetes in China: Epidemiology and Genetic Risk Factors and Their Clinical Utility in Personalized Medication. Diabetes.

[11] Pan J, Jia W (2018). Early-onset diabetes: an epidemic in China. Front Med.

[12] Kasiya MM, Mang'anda GD, Heyes S, Kachapila R, Kaduya L, Chilamba J (2017). The challenge of diabetic foot care: Review of the literature and experience at Queen Elizabeth Central Hospital in Blantyre, Malawi. Malawi Med J.

[13] Alavi A, Sibbald RG, Mayer D, Goodman L, Botros M, Armstrong DG, et al. Diabetic foot ulcers Part I. Pathophysiology and prevention. J Am Acad Dermatol. 2014;70(1). 10.1016/j.jaad.2013.06.055 PMID:24355275. 10.1016/j.jaad.2013.06.055 24355275

[14] Jordan DN, Jordan JL (2011). Foot self-care practices among Filipino American women with type 2 diabetes mellitus. Diabetes Ther.

[15] Al Sayah F, Soprovich A, Qiu W, Edwards AL, Johnson JA (2015). Diabetic Foot Disease, Self-Care and Clinical Monitoring in Adults with Type 2 Diabetes: The Alberta's Caring for Diabetes (ABCD) Cohort Study. Can J Diabetes.

[16] Walsh JW, Hoffstad OJ, Sullivan MO, Margolis DJ (2016). Association of diabetic foot ulcer and death in a population-based cohort from the United Kingdom. Diabet Med.

[17] Inker LA, Astor BC, Fox CH, Isakova T, Lash JP, Peralta CA (2014). KDOQI US Commentary on the 2012 KDIGO Clinical Practice Guideline for the Evaluation and Management of CKD. Am J Kidney Dis.

[18] Schaper NC (2004). Diabetic foot ulcer classification system for research purposes: a progress report on criteria for including patients in research studies. Diabetes Metab Res Rev.

[19] Pickwell K, Siersma V, Kars M, Apelqvist J, Bakker K, Edmonds M (2015). Predictors of Lower-Extremity Amputation in Patients With an Infected Diabetic Foot Ulcer. Diabetes Care.

[20] Chuan F, Tang K, Jiang P, Zhou B, He X. Reliability and Validity of the Perfusion, Extent, Depth, Infection and Sensation (PEDIS) Classification System and Score in Patients with Diabetic Foot Ulcer. Plos One. 2015;10(4). 10.1371/journal.pone.0124739 PMID:25875097. 10.1371/journal.pone.0124739 PMC4395335 25875097

[21] Yan J, Liu Y, Zhou B, Sun M (2014). Pre-hospital delay in patients with diabetic foot problems: influencing factors and subsequent quality of care. Diabet Med.

[22] Chu Y-J, Li X-W, Wang P-H, Xu J, Sun H-J, Ding M (2016). Clinical outcomes of toe amputation in patients with type 2 diabetes in Tianjin, China. Int Wound J.

[23] Margolis DJ, Allen-Taylor L, Hoffstad O, Berlin JA (2002). Diabetic neuropathic foot ulcers: the association of wound size, wound duration, and wound grade on healing. Diabetes Care.

[24] Monami M, Longo R, Desideri CM, Masotti G, Marchionni N, Mannucci E (2008). The diabetic person beyond a foot ulcer - Healing, recurrence, and depressive symptoms. J Am Podiatr Med Assoc.

[25] Winkley K, Stahl D, Chalder T, Edmonds ME, Ismall K (2007). Risk factors associated with adverse outcomes in a population-based prospective cohort study of people with their first diabetic foot ulcer. J Diabetes Complications.

[26] Pineda J, Marin F, Roldan V, Valencia J, Marco P, Sogorb F (2008). Premature myocardial infarction: Clinical profile and angiographic findings. Int J Cardiol.

[27] Chua S-K, Hung H-F, Shyu K-G, Cheng J-J, Chiu C-Z, Chang C-M (2010). Acute ST-elevation Myocardial Infarction in Young Patients: 15 Years of Experience in a Single Center. Clin Cardiol.

[28] Reinstadler SJ, Eitel C, Thieme M, Metzler B, Poess J, Desch S (2016). Comparison of Characteristics of Patients aged <= 45 Years Versus > 45 Years With ST-Elevation Myocardial Infarction (from the AIDA STEMI CMR Substudy). Am J Cardiol.

[29] McEwen LN, Ylitalo KR, Herman WH, Wrobel JS (2013). Prevalence and risk factors for diabetes-related foot complications in Translating Research Into Action for Diabetes (TRIAD). J Diabetes Complications.

[30] Sibbald RG, Woo KY (2008). The biology of chronic foot ulcers in persons with diabetes. Diabetes Metab Res Rev.

[31] Berlanga-Acosta J, Schultz GS, Lopez-Mola E, Guillen-Nieto G, Garcia-Siverio M, Herrera-Martinez L (2013). Glucose toxic effects on granulation tissue productive cells: the diabetics' impaired healing. Biomed Res Intl.

[32] Huo X, Gao L, Guo L, Xu W, Wang W, Zhi X (2016). Risk of non-fatal cardiovascular diseases in early-onset versus late-onset type 2 diabetes in China: a cross-sectional study. Lancet Diabetes Endocrinol.

[33] Zubair M, Malik A, Ahmad J (2011). The impact of creatinine clearance on the outcome of diabetic foot ulcers in north Indian tertiary care hospital. Diabetes Metab Syndr.

[34] Bonifant H, Holloway S (2019). A review of the effects of ageing on skin integrity and wound healing. Br J Community Nurs.

[35] Hokkam EN (2009). Assessment of risk factors in diabetic foot ulceration and their impact on the outcome of the disease. Prim Care Diabetes.

[36] Jiang Y, Ran X, Jia L, Yang C, Wang P, Ma J (2015). Epidemiology of Type 2 Diabetic Foot Problems and Predictive Factors for Amputation in China. Int J Low Extrem Wounds.

[37] Schmaltz HN, Southern D, Ghali WA, Jelinski SE, Parsons GA, King KM (2007). Living alone, patient sex and mortality after acute myocardial infarction. J Gen Intern Med.

[38] Nielsen FE, Mard S (2010). Single-living is associated with increased risk of long-term mortality among employed patients with acute myocardial infarction. Clin Epidemiol.

[39] Vujcic I, Vlajinac H, Dubljanin E, Vasiljevic Z, Matanovic D, Maksimovic J (2015). Long-term prognostic significance of living alone and other risk factors in patients with acute myocardial infarction. Ir J Med Sci.

